# Synovial Matrix Remodeling and Inflammatory Profile in Disc Displacement of the Temporomandibular Joint: An Observational Case-Control Study

**DOI:** 10.1155/2024/2450066

**Published:** 2024-09-18

**Authors:** Pallavi Khattar, Mattias Ulmner, Henrike Häbel, Bodil Lund, Rachael V. Sugars

**Affiliations:** ^1^ Department of Dental Medicine Karolinska Institutet, Stockholm 171 77, Sweden; ^2^ Medical Unit of Plastic Surgery and Oral and Maxillofacial Surgery Karolinska University Hospital, Stockholm 171 76, Sweden; ^3^ Department of Learning, Informatics, Management and Ethics Karolinska Institutet, Stockholm 171 77, Sweden

## Abstract

**Background:** Pain-related temporomandibular joint disorders (TMJD) are a major public health problem, including the diagnoses of disc displacement (DD) with and without reduction (DDwR/DDwoR).

**Objectives:** The study aimed to examine the matrix remodeling and the inflammatory profile in synovial tissues of patients with TMJ-DD, with a view to understand the pathophysiology, and to contribute to the development of tissue-based diagnostic criteria.

**Methods:** This laboratory-based observational case-control study included 30 synovial tissue samples obtained from 30 patients, diagnosed with delayed (DO) or sudden (SO) onset of DDwoR, which were compared against the reference patient material, DDwR (*n* = 10/diagnosis group). Tissue samples were investigated histologically and via quantitative immunohistochemistry for a panel of antibodies targeted against extracellular matrix proteins and inflammatory markers. The data were analyzed using a generalized linear model with a gamma family distribution (*p* < 0.05).

**Results:** Quantification of immunostaining revealed significant differences in the distribution of collagen type III (DO, *p* < 0.001), lumican (DO, *p* < 0.05), matrix metalloproteinase-2 (DO, *p* < 0.05), CD4 T-helper cells (DO, *p* < 0.01; SO, *p* < 0.001), and CD68 monocytic immune cells (both SO and DO, *p* < 0.001) in DDwoR groups compared to the reference patient material, DDwR.

**Conclusions:** The observations confirmed differences in matrix remodeling and an increase in local inflammatory activity in the DDwoR diagnosis compared to the reference patient material, DDwR. The study highlighted the importance of synovial tissue characterization to unite micropathology and clinical findings, leading to more reliable diagnostic tools.

## 1. Introduction

Temporomandibular joint disorders (TMJD) refer to structural aberrations within the joint associated with pain and impairment of functional capacity [1, 2]. The prevalence of TMJD is ~31% for adults/elderly and 11% for children/adolescents [3].

Disc displacement (DD) is the most common TMJD with an overall prevalence of ~19% and is described as a sequentially developing disease [2, 3]. The disorder is subdivided into DD with and without reduction (DDwR/DDwoR) [2, 4]. DDwR occurs when the articular disc dislocates relative to the condylar head and momentarily repositions during mouth opening [3, 5, 6]. Studies have consistently shown fibrotic changes in DDwR tissues, with no or minor synovial inflammation [5, 6]. DDwoR is a degenerative disorder accompanied by inflammation of the synovial tissues, in which the articular disc remains in the displaced position [2, 5, 6]. Clinical observations might suggest a further division of DDwoR as delayed (DO) or sudden (SO) onset [2, 6–8]. DDwoR-DO is preceded by a history of DDwR, while DDwoR-SO lacks any previous DD symptoms [2, 6–8].

Extracellular matrix (ECM) is a dynamic structure, rich in collagen that continuously undergoes controlled remodeling [9]. However, any dysregulation of its composition may contribute to pathological conditions, such as fibrosis [9]. Matrix metalloproteinases (MMPs) facilitate ECM turnover and are regulated by endogenous natural tissue inhibitors of MMPs (TIMPs) [10]. MMPs play a key role in wound healing via the regulation of chemokine activity and immune responses, for instance, the activation of transforming growth factor-beta (TGF-*β*), and it has been speculated that MMP−2 and TIMP−2 are involved in TMJ-DD [10–12]. TGF-*β* is an important mediator of tissue repair and regulator of ECM turnover [10, 13, 14]. TGF-*β* isoforms exert context-dependent effects on wound healing [14]. TGF-*β*1, the most prevalent isoform, is very potent and acts pro-fibrotically, while TGF-*β*3 may promote scarless healing and antifibrotic effects [14, 15]. In vivo studies have suggested that mechanical forces, such as the physiological shearing of synovial fluid, may promote TGF-*β* activation [13, 16]. Lumican, a small leucine-rich proteoglycan has a role in inflammation and maintenance of tissue structural homeostasis, as well as collagen fibrillogenesis [17, 18]. Lumican has been demonstrated in both healthy and degenerative joint disease (DJD) affected synovial tissues, where it has been localized to synovial surface areas subjected to increased mechanical stress [19, 20]. Although data on lumican involvement in TMJD is limited, studies have suggested a role in early stages of inflammation and promotion of temporomandibular joint (TMJ) regeneration following interleukin-1 beta induced degeneration [17, 20].

ECM remodeling and inflammation in TMJD synovial tissues have been revealed clinically and histopathologically [21–24]. Studies investigating inflammatory cell occurrence in retrodiscal tissues demonstrated T-cell dominated slight inflammation and few macrophages in DDwR [22]. In contrast, DJD biopsies showed frequent macrophages, with a more complex cytokine profile [22]. However, detailed investigations are warranted to understand DD pathophysiology [7, 8, 25].

The current knowledge in this field remains limited, with matrix remodeling and immune cell profiling sparsely investigated in TMJD synovial tissues [5, 22]. A major hindrance to such studies is partially due to ethical constrictions and the lack of comparison against healthy tissue samples. Secondly, the production and concentration of proteins in synovial tissue biopsies might describe the local status, for instance, the degree of inflammation and degradation, more precisely compared to synovial fluid [26]. Synovial tissue-based diagnostics might consequently interlink micropathology and clinical findings, resulting in more reliable diagnostic and treatment tools [8, 22, 25]. Therefore, this study examined fibrosis, matrix remodeling patterns, and the inflammatory profile of synovial tissue biopsies from patients with TMJ-DD. A secondary aim was to scrutinize the subdiagnosis DDwoR of DO and SO and eventual differences.

## 2. Materials and Methods

### 2.1. Study Design

This laboratory-based observational case-control study was performed at the Department of Dental Medicine, Karolinska Institutet, Stockholm, Sweden. The study was approved by the Swedish Ethical Review Authority (reference number 2014/622-31/1) [8]. Written informed consent was obtained from all participants, and patients were given the right to withdraw at any time without consequences. The study complied with the STROBE guidelines [27].

### 2.2. Study Population

The inclusion period was from December 2014 to January 2017 [8, 25]. All participants met the following inclusion criteria: patients diagnosed with DDwR and DDwoR following the diagnostic criteria for temporomandibular disorders (DC/TMD), and previous conservative treatment approaches tried for at least 3 months [4, 8, 25]. Exclusion criteria were patients aged under 18 years, lack of informed consent and patients who had undergone prior open surgery of the TMJ [8, 25]. Patients diagnosed with DDwoR were further subdiagnosed into the groups DDwoR-DO and DDwoR-SO after careful evaluation of anamnestic findings. DO represents the classical DDwoR, where the patient described previous popping and clicking from the affected joint (DDwR) which with time developed into DDwoR. SO was diagnosed when the patient described their TMJ as being earlier symptom-free with a swift deterioration and signs of pain and limited mouth-opening capacity.

**Figure 1:**
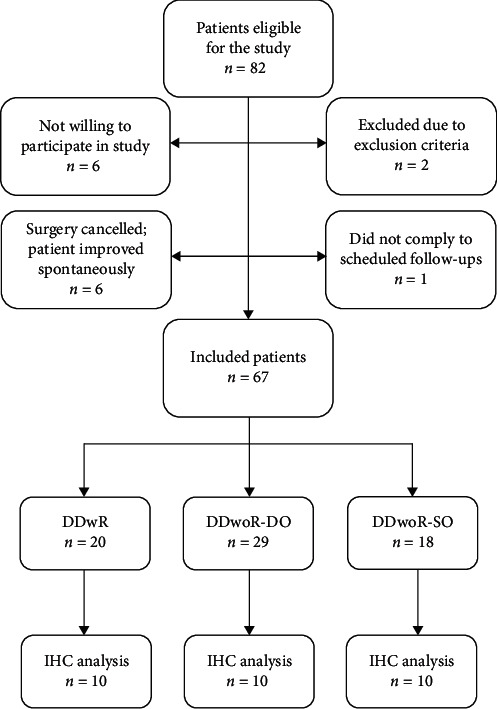
Flowchart illustrating patient's eligibility for inclusion into the study. Ten patients were consecutively withdrawn from each subgroup. DDwR, disc displacement with reduction; DDwoR, disc displacement without reduction; DO, delayed onset; IHC, immunohistochemistry; SO, sudden onset.

The patient samples consisted of synovial tissue biopsies from the superior aspect of the posterior bilaminar zone [8, 25]. The 30-patient cohort was consecutively drawn from a total of 67 patients who had been included following a power calculation and carefully described in the previous research [8, 25]. The first 10 patients from each diagnostic group (DDwoR-DO, *n* = 10; DDwoR-SO, *n* = 10; DDwR, *n* = 10) were chosen for analyses (Figure 1) [8]. Due to ethical considerations, no TMJ healthy patients could be enrolled as controls. Instead, patients with DDwR were used as reference material, as this patient group has previously been identified with no or very mild inflammation and no degenerative changes [2, 6, 22].

### 2.3. Surgical Procedure and Collection of Tissue Samples

The operative interventions, arthroscopic lysis and lavage and discectomy, were performed by MU at the Medical Unit of Plastic Surgery and Oral and Maxillofacial Surgery, Karolinska University Hospital, Stockholm, Sweden. Following the clinical sampling, the biopsies were fixed in 4% paraformaldehyde, paraffin-embedded, and sectioned.

### 2.4. Histological Staining

Two 4 µm thick paraffin-embedded synovial tissue sections were mounted on each slide (Super Frost Plus slides; Menzel-Gläser, Braunschwig, Germany). Deparaffinised and rehydrated tissues were histologically stained with Mayer's Hematoxylin Plus (HTX; Histolab Products AB, Gothenburg, Sweden) and Eosin (Histolab Products AB) for basic tissue morphology, Trichrome Stain (Masson) Kit (Sigma–Aldrich, Stockholm, Sweden) to visualize connective tissues, and van Gieson (Sigma–Aldrich) to assess the degree of fibrosis. The sections were dehydrated through a graded series of alcohols into xylene and mounted in Pertex (Histolab Products AB).

### 2.5. Immunohistochemistry

Deparaffinised rehydrated sections were treated with heat antigen epitope retrieval using basic buffer pH 9 (R&D Systems, Cambridge, United Kingdom) for antibodies against CD4 (Abcam, Cambridge, United Kingdom) and CD68 (DAKO, Golstrup, Denmark), and citrate buffer pH 6 (Thermo Fisher Scientific, Waltham, Massachusetts, USA) for MMP-2 (Abcam), TIMP-2 (Abcam), lumican (R&D Systems), and collagens types I and III (Abcam). To expose TGF-*β*1 (Santa Cruz Biotechnology (SCBT) Inc., United States) and TGF-*β*3 (SCBT) epitopes, sections were incubated with 0.2M hydrochloric acid (HCl). Nonspecific background staining was reduced using endogenous peroxidase with 3% (v/v) hydrogen peroxide and blocking of Fc-receptors and tissue permeabilization with 10% normal serum (NGS/NRS; DAKO) and 0.3% Triton X-100 (Sigma–Aldrich). Primary monoclonal mouse antihuman (TIMP-2 1 : 300; collagen type III 1 : 3000 and CD68 1 : 750), monoclonal rabbit antihuman (collagen type I 1 : 3000 and CD4 1 : 1000), polyclonal rabbit antihuman IgG (TGF-*β*1 1 : 500 and TGF-*β*3 1 : 150), polyclonal goat antihuman IgG (MMP-2 1 : 1000 and lumican 1 : 800) antibodies were diluted in 4% normal serum and incubated at 4°C overnight. Positive and negative controls were included to ensure the validity of the staining (Supporting Information S2: Data 2). Biotinylated secondary antibodies (goat antirabbit-IgG, goat antimouse-IgG, or rabbit antigoat-IgG; 1 : 500; Vector Laboratories, Burlingame, United States) were incubated for 1 h at room temperature. Preincubated ABC Elite Kit Reagent (Vector Laboratories) was applied to the sections for 30 min followed by detection with DAB (3,3´-diaminobenzidine substrate; liquid DAB + substrate chromogen system, DAKO) with optimized development times for each antibody. Slides were counterstained with Mayer's Hematoxylin (Histolab Products AB), followed by dehydration and mounting in Pertex (Histolab Products AB). All rinses were in tris-buffered saline (50 mM tris, 150 mM sodium chloride, pH 7.6) with 0.1% Tween 20 (TBST; Sigma–Aldrich).

### 2.6. Digitalization and Image Analysis

The stained slides were scanned and digitalized to obtain whole slide images using the 3D Histech Midi Scanner System and viewed in CaseViewer Software 2.4 (3D Histech, Histolab Products AB). Images were annotated, exported, and segmented into 1500 × 1500 pixels (> 15 kb) using ImageMagick Software version 7.0.8 (www.imagemagick.org). Immunohistochemical (collagen types I and III, lumican, MMP-2, TIMP-2, TGF-*β*1 and -*β*3 for ECM remodeling and CD4, CD68 for immune profiling) and histological staining (van Gieson for fibrosis) quantification was performed using the open-access image analysis Cell-Profiler Software version 4.2.1 (www.cellprofiler.org) [28]. A workflow was optimized in CellProfiler for each antibody/stain through manual inspection, in which the thresholds were adjusted to assess specific staining [28]. The workflows are available through https://cellprofiler.org/published-pipelines or upon request [28]. Tiles with artifacts or folds were manually excluded prior to data analysis. The data outputs for fibrosis and total chromogenic (DAB) stained pixel area per total pixel area, were extracted as Excel files, and the data recombined to determine the average per biopsy [28].

### 2.7. Statistical Analysis

Analyses were conducted in separate groups for each antibody/stain, with respect to the ratio between DAB positive or van Gieson pixel area over total pixel area [28]. Statistical analyses were performed using generalized linear model with a gamma distribution, as the pixel area data showed a positively skewed continuous distribution in each group. The results were reported as fold change (marginally predicted mean pixel area ratios) for all groups, and the DDwoR-DO and DDwoR-SO groups were compared against the reference patient material, DDwR, which was normalized to 1. Postestimation pairwise comparisons between individual groups were performed, followed by Bonferroni correction to reduce familywise error rate. Data analyses were performed with Stata version 16 (StataCorp, College Station, TX). 95% confidence intervals are presented, with statistical significance considered at *p* < 0.05.

## 3. Results

### 3.1. Patient Demographic Data

This laboratory-based observational case-control study included 30 synovial tissue biopsies incised from patients undergoing either arthroscopy or discectomy (Table 1). One patient from the DDwR subgroup was excluded based on mixed clinical symptoms that could not be assigned a specific subdiagnosis. This resulted in the final cohort of 29 patients; nine in DDwR, 10 in DDwoR-DO, and 10 in DDwoR-SO (Table 1).

### 3.2. Histological Staining

**Figure 2:**
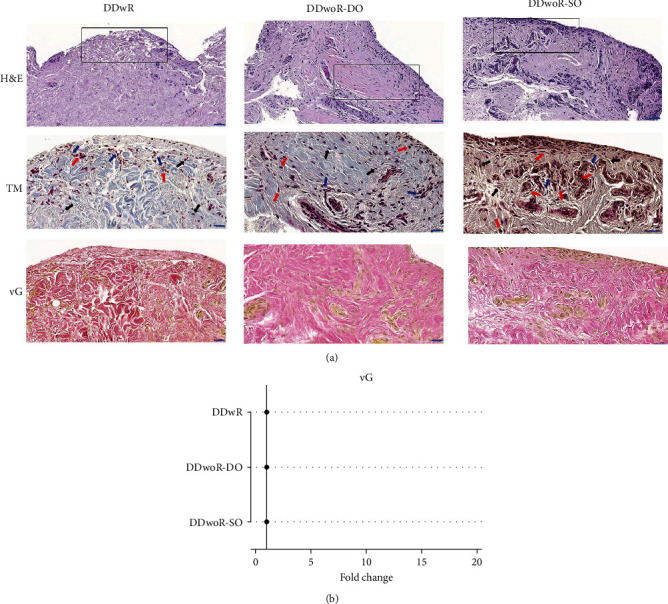
Histological staining and quantification of fibrosis in synovial tissues from patients with disc displacement of the temporomandibular joint. (a) Representative images showing histological staining (hematoxylin and eosin, Trichrome Masson, and van Gieson) of synovial tissues from patients with DDwR, DDwoR-DO, and DDwoR-SO, respectively. Hematoxylin and Eosin images at lower magnification (scale bar 50 µm) provide a general overview of the synovial biopsies. The box denotes region of focus for subsequent images. The higher magnification images for Trichrome Masson and van Gieson (scale bar 20 µm) feature basic cellular and tissue structure. The biopsies from both DDwR and DDwoR were rich in fibrous tissues, containing resident fibroblasts (black arrows), macrophages (red arrows), and infiltrating (blue arrows) cells in a collagenous extracellular matrix (highlighted on the Trichrome Masson stain). (b) Quantification of fibrosis staining, performed using generalized linear models with a gamma distribution, did not demonstrate any significant changes in mean pixel area ratio (fold change and 95% confidence interval) between the groups (Supporting Information S1: Data 1). The dotted lines indicate the normalized value (1) for DDwR group from which the comparisons were made. DDwR, disc displacement with reduction; DDwoR, disc displacement without reduction; DO, delayed onset; H&E, hematoxylin and eosin; SO, sudden onset; TM, Trichrome Masson; vG, van Gieson.

Histological staining provided a general overview of the synovial biopsies' basic cellular and tissue structure. Overall, fibrous synovial tissues containing resident fibroblasts, macrophages, and infiltrating cells, in a collagenous ECM with scattered blood vessels and fat cells were observed (Figure 2a). Lymphocytic cells were mostly present near the blood vessels but also appeared randomly distributed between fibers further away from the vessels (Figure 2a). The appearance of the synovium varied considerably, not only between the three subdiagnoses groups but also within each group.

Van Gieson staining was used to assess the degree of fibrosis within the tissues (Figure 2a). Both DDwR and DDwoR specimens were rich in fibrous tissues; however, DDwR tissues appeared to have a slightly more amorphous-like structure, with a collagen-rich ECM of light pink-orange color, while DDwoR tissues had distinctive, red-colored collagen fibers (Figure 2a). Despite considerable inter- and intragroup variations in the morphological tissue structure, the distribution of fibrosis was comparatively similar across all three tissue types. Accordingly, there were no statistically significant differences regarding the quantification of fibrosis between the groups (Figure 2a, Supporting Information S1: Data 1).

### 3.3. ECM Distribution and Turnover

**Figure 3:**
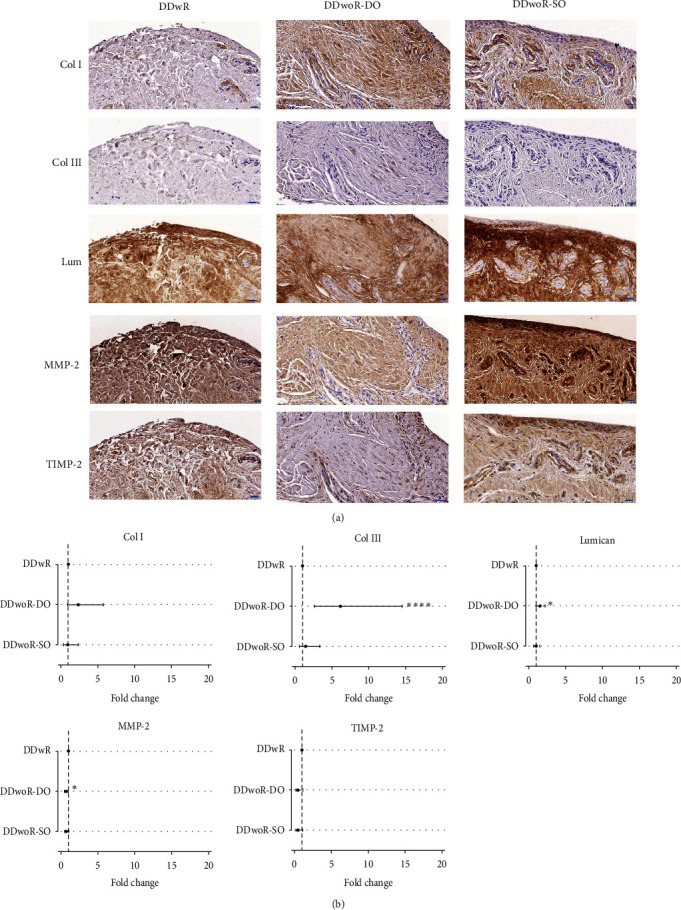
Immunohistochemical staining and quantification of extracellular matrix components in synovial tissues from patients with disc displacement of the temporomandibular joint. (a) Images show immunohistochemical localization for collagen types I and III, lumican, MMP-2, TIMP-2, from patients with DDwR, DDwoR-DO, and DDwoR-SO (scale bars are 20 µm). (b) Quantification of immunohistochemical staining was performed using generalized linear models with a gamma distribution and demonstrated significant changes in mean pixel area ratio (fold change and 95% confidence interval) between DDwR and the two DDwoR subdiagnoses, DO and SO (Supporting Information S1: Data 1). The dotted lines indicate the normalized value (1) for DDwR group from which the comparisons were made. DDwR, disc displacement with reduction; DDwoR, disc displacement without reduction; DO, delayed onset; Lum, lumican; MMP-2, matrix metalloproteinase-2; SO, sudden onset; TIMP-2, tissue inhibitor of metalloproteinase-2.  ^*∗∗∗∗*^*p* ≤ 0.001 and  ^*∗*^*p* ≤ 0.05.

Widespread staining for collagen type I and lumican was observed across all three diagnostic groups, but the distribution of collagen type III was scarce (Figure 3a). Collagen type I fibers were located throughout the tissues, especially surrounding blood vessels, and were generally more aligned in DDwR tissues. Collagens types I and III and lumican were more frequently distributed in DDwoR-DO tissues (Figure 3a). MMP-2 was visible in association with fibrous fields. Areas near vessels showed no staining for MMP-2 (Figure 3a). TIMP-2 distribution was less abundant than MMP-2 and, in contrast to MMP-2, TIMP-2 staining was condensed in the cell bodies around capillaries. DDwR tissues, in comparison to DDwoR subgroups, demonstrated more abundant staining for both MMP-2 and TIMP-2.

DDwoR-DO, MMP-2 staining, significantly decreased 1.5-fold (*p* < 0.05, CI 2-fold to 1-fold) in comparison to DDwR but was elevated 6-fold (*p* < 0.001, CI 3-fold to 15-fold) and 1.5-fold (*p* < 0.05, CI 1-fold to 2-fold) for collagen type III and lumican, respectively (Figure 3b; Supporting Information S1: Data 1). Postestimation pairwise comparisons using Bonferroni correction showed further statistically significant differences in distribution between collagen type III and both DDwoR-DO and -SO (*p* < 0.05), and DDwR (*p* < 0.05) (Supporting Information S1: Data 1). No statistically significant differences were seen for collagen type I and TIMP−2 between the diagnoses (Figure 3b; Supporting Information S1: Data 1).

**Figure 4:**
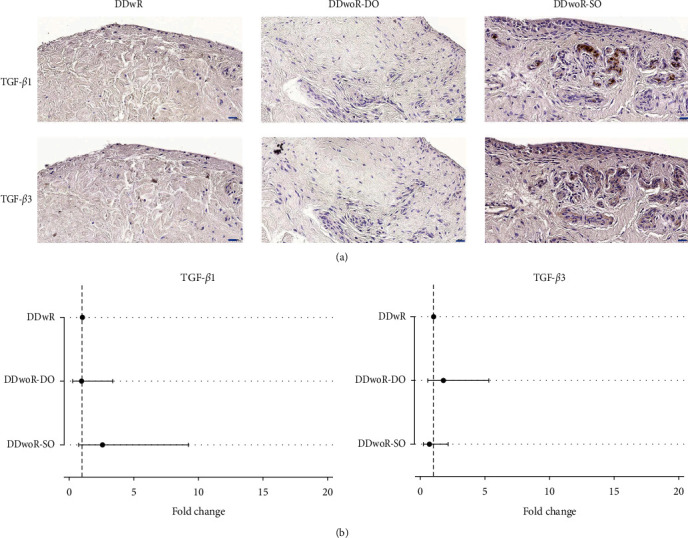
Immunohistochemical staining and quantification of transforming growth factor-beta 1 and 3 in synovial tissues from patients with disc displacement of the temporomandibular joint. (a) Images show immunohistochemical localization for TGF-*β*1 and -*β*3 from patients with DDwR, DDwoR-DO, and DDwoR-SO (scale bars are 20 µm). (b) Quantification of immunohistochemical staining, performed using generalized linear models with a gamma distribution, showed no significant changes in mean pixel area ratio (fold change and 95% confidence intervals) between the groups (Supporting Information S1: Data 1). The dotted lines indicate the normalized value (1) for DDwR group from which the comparisons were made. DDwR, disc displacement with reduction; DDwoR, disc displacement without reduction; DO, delayed onset; SO, sudden onset; TGF-*β*1 and -*β*3, transforming growth factor-beta 1 and 3.

TGF-*β*1 was localized in the ECM, and aggregations were found in close association with the blood vessels (Figure 4a). TGF-*β*3 was condensed in the cell bodies associated with endothelial and perivascular cells, and layers surrounding capillaries, with little staining within the ECM (Figure 4a). Both isoforms followed this general pattern of distribution across the three diagnoses. DDwoR-DO presented a slightly larger distribution of chromogenic staining for TGF-*β*3 and DDwoR-SO for TGF-*β*1 than their corresponding groups; however, fold changes were not significant (Figure 4b; Supporting Information S1: Data 1).

### 3.4. Inflammatory Profile

**Figure 5:**
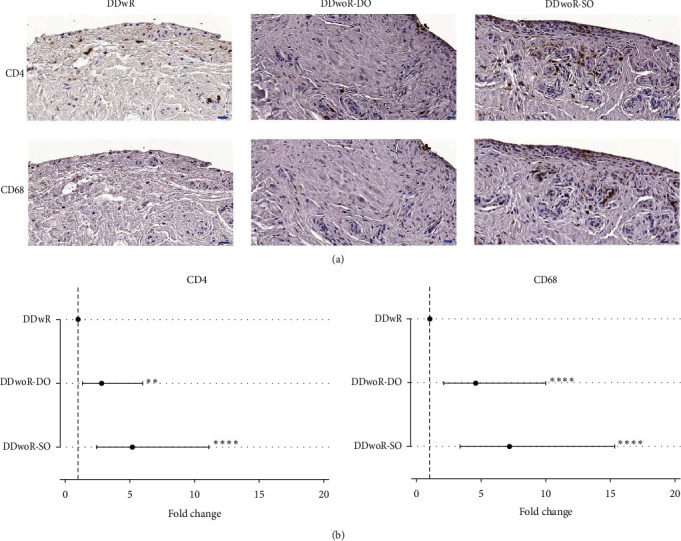
Immunohistochemical staining and quantification of immune cells in synovial tissues from patients with disc displacement of the temporomandibular joint. (a) Images show immunohistochemical localization for CD4 T-helper and CD68 monocytic immune cells, in patients with DDwR, DDwoR-DO, and DDwoR-SO, respectively. Patients with DDwoR-SO group demonstrated a larger distribution of chromogenic staining for CD4 and CD68 positive cells, suggestive of increased degree of inflammation, whereas DDwR group had noticeably less amount of chromogenic staining (scale bars are 20 µm). (b) Quantification of immunohistochemical staining was performed using generalized linear models with a gamma distribution and demonstrated significant changes in mean pixel area ratio (fold change and 95% confidence intervals) between DDwR and the two DDwoR subdiagnoses, DO and SO (Supporting Information S1: Data 1). The dotted lines indicate the normalized value (1) for DDwR group from which the comparisons were made. CD, cluster of differentiation; DDwR, disc displacement with reduction; DDwoR, disc displacement without reduction; DO, delayed onset; SO, sudden onset.  ^*∗∗∗∗*^*p* ≤ 0.001 and ^*∗∗*^*p* ≤ 0.01.

The aggregations of CD4 T-helper cells and CD68 monocytic cells were observed throughout the tissues (Figure 5a). However, these cells were more frequent near the blood vessels and only occasionally observed in association with the fibrous regions. CD68 cells were present in both the intimal and subintimal layers, while the CD4 cells were slightly more frequent in the subintimal regions (Figure 5a). Visual assessment and statistical analyses confirmed a more frequent distribution of CD4 and CD68 cells in the DDwoR tissues, in comparison to DDwR (Figures 5a,b). For DDwoR-DO, CD4 immunolocalization significantly increased 3-fold (*p* < 0.01, CI 1-fold to 6-fold) and CD68 5-fold (*p* < 0.001, CI 2-fold to 10-fold), against DDwR tissues (Figure 5b; Supporting Information S1: Data 1). Whereas for DDwoR-SO tissues, the CD4 profile increased to 5-fold (*p* < 0.001, CI 2-fold to 11-fold) and CD68 to 7-fold (*p* < 0.001, CI 3-fold to 15-fold), compared to the reference DDwR material (Figure 5b; Supporting Information S1: Data 1). Postestimation pairwise comparison using Bonferroni's correction showed statistically significant differences for CD4 between DDwR and DDwoR-SO (*p* < 0.01) and CD68 between both DDwR, and DDwoR-DO (*p* < 0.05), and DDwoR-SO (*p* < 0.005) (Supporting Information S1: Data 1).

## 4. Discussion

Pain-related TMJD is a major public health problem, causing a considerable impact on an individual's quality of life due to functional pain, psychosocial disturbances, and hindrance in daily life activities [3, 29, 30]. The aetiopathogenesis is complex and obscure, and one of the remaining methodological problems is the accurate definition of the applied criteria [3, 31]. Previous research in this field has repeatedly indicated the need for synovial tissue-based diagnostics, derived from fundamental comprehension of the basic disease pathology, and underlying biological processes [22, 26]. Therefore, we aimed to examine the matrix composition and remodeling, and inflammatory cell activity in the synovial tissues in patients with TMJ-DD to understand the micropathology, and to contribute to the development of tissue-based diagnostic criteria. To our knowledge, this is the first study to compare the two DD diagnoses with a large panel of antibodies, targeted against ECM proteins and inflammatory markers, via quantitative immunohistochemistry, in synovial tissue biopsies. The outcomes of our research, thereby, not only support previous knowledge on DD diagnoses but also urges the need to further develop the diagnostic criteria.

In healthy tissues, the highly dynamic ECM continuously undergoes controlled remodeling and any dysregulation of the ECM might contribute to pathological conditions, such as fibrosis [9]. A DDwR diagnosis has been associated with fibrotic changes, as a result of repetitive trauma to the articular disc, but the simultaneous occurrence of fibrosis and chronic inflammation, as in DDwoR, has also been demonstrated with degenerative changes, synovial proliferation, and production of adhesions [2, 5]. Our quantification of fibrosis, and distribution of collagen type I, did not present any statistically significant differences between DDwR and DDwoR diagnoses. Both groups showed a broad distribution of fibrosis, collagen type I, and lumican, in contrast to, collagen type III which was less frequent. Elevated collagen type III is mainly associated with scar tissue formation, where thinner fibrils form an initial randomly oriented network that is replaced over time by a stronger and better-aligned network of collagen type I fibers [32]. The longstanding repetitive mechanical stress and repair cycle, in DDwoR-DO tissues, could support the increased immunolocalization of both collagen type III and lumican [19, 20]. An upregulation of lumican in areas with increased mechanical stimuli has been reported in other studies, which suggests that lumican may function in the early stages of inflammation and promote regeneration of the TMJ, following interleukin-1 beta induced degeneration [17, 20].

The interplay between MMPs and TIMPs is known to regulate ECM remodeling and wound healing processes, with MMP-2 reported to be crucial in tissue fibrosis [33, 34]. The distribution of both MMP-2 and TIMP-2 was reduced in DDwoR compared to the DDwR group, with a statistically significant decrease for MMP-2 in the DDwoR-DO subgroup. A previous investigation, with a small sample size, indicated an association between increased MMP-2 levels and a more severe TMJ disorder [35]. The study analyzed MMP-2 immunostaining and, contrary to our results, showed significantly increased levels of MMP-2 in DDwoR compared with DDwR tissues [35]. Interestingly, our results reflect the increased expression of MMPs during the early stages of fibrotic change in DDwR, and a reduction of MMP-2 after the recovery stage, representing DDwoR-DO [33]. These findings allow the speculation that DDwR may develop into DDwoR-DO [36]. This also gives rise to another hypothesis involving the time-dependent adaptation process in cases where patients with any kind of DD might recover by the formation of a pseudo-disc, without any need for interventions [10, 37, 38].

Active TGF-*β* is present in normal physiological conditions, albeit in small quantities, as seen in our DDwR reference material [39]. In the current study, no statistically significant fold changes of the two TGF-*β* isoforms were observed between the DD diagnoses. However, the results indicated a slight increase of TGF-*β*1 in DDwoR-SO tissues and TGF-*β*3 in DDwoR-DO tissues. Our knowledge regarding TGF-*β*1 distribution in synovial tissues is limited, although the pro-fibrotic role of TGF-*β*1, compared to TGF-*β*3 antifibrotic effects and ability to stimulate scarless healing, suggests different mechanisms of action [14, 15]. Larkin et al. [40] proposed a time and disease-stage-dependent localization of TGF-*β*. Early onset pathological conditions, like DJD and fractures, initiate short-term elevated TGF-*β*1 bioactivity, and upregulation of collagen type I, leading to increased fibrosis [23, 39]. Subsequently, TGF-*β*1 expression is described to be almost absent in later stages of disease progression [40–42]. The upregulation of DDwoR anabolic effects, in tissues, is probably indicative of a simultaneous healing response to injured tissue rather than a primary alteration as reported in a number of studies [22, 23, 39, 42, 43]. The complex cytokine pattern in DJD, reported by Kardel et al. [22, 23], involving also anti-inflammatory cytokines, such as TGF-*β*, was attributed to simultaneous repair mechanisms, which correlated to arthroscopic findings of tissue destruction and repair. High levels of TGF-*β*1 have also been related to increased osteophyte formation and synovial fibrotic features of DJD [41]. Despite correlations between TGF-*β* and inflamed synovia, studies could not associate its presence with enhanced cartilage degradation [39].

The DDwoR tissues were significantly associated with both CD4 and CD68 staining, compared to the DDwR reference material, indicating a higher inflammatory activity in the former group. Similar investigations, using antibodies against CD45RO T-memory cells and CD68 cells, to compare DDwR to DJD, demonstrated T-cell dominated slight inflammation, with few macrophages in DDwR specimens [22, 44]. Furthermore, the outcomes suggested an important role of CD68 monocytic cells in the DJD [22, 40, 44–46]. In our study, similar results were found between DDwR and DDwoR groups. To emphasize, DD might be a sequentially developing disease and according to Wilkes [2] DD staging criteria, DDwR might precede the development of DDwoR-DO. We encountered problems while categorizing patients with mixed clinical symptoms, and consequently, one patient was excluded from the study. Patients suffering from DD can also develop DJD [2].

There were no significant differences in the distribution of the immune cell markers between the two DDwoR groups, although the difference in fold change still indicated an elevated immunostaining of both antibodies in DDwoR-SO tissues. A possible reason may perhaps be that all patients underwent conservative treatment approaches prior to surgical intervention, aiming to relieve pain and reduce inflammation, introducing a potential confounding factor [1]. Previously, analyses of the DDwoR-SO subgroup reported higher concentrations of bone morphogenetic protein-4, eotaxin, and interleukin-8 compared to DDwoR-DO [8]. Interleukin-8 stimulates phagocytosis, which may contribute to the significantly higher levels of CD68 and CD4 cells in both DDwoR subgroups [8, 47]. This gives rise to speculations that patients in the DDwoR-SO group might be predisposed to an early stage of DJD, perhaps representing an independent entity [2, 7, 8]. Also, the increased frequency of trauma, in the DDwoR-SO group warrants further studies to clarify and determine whether it is an inciting event, or merely a cumulative factor, in the development of DD [8–48, 48–51].

This study had a few limitations. The patient cohort had an apparent skewed gender distribution toward a stronger female predominance (9 : 1 ratio), which corresponded to preceding TMJD studies [5, 10, 52, 53]. Its greater frequency and severity in females have been attributed to biological, psychosocial, hormonal, and cultural factors [52–54]. For ethical reasons, surgical specimens cannot be obtained from healthy controls; however, previous research suggests that DDwR specimens can be used as reference material in studies primarily focused on degenerative and inflammatory joint disease [5, 6, 22, 55]. Furthermore, the limited sample size and lack of previous studies investigating similar variables using synovial tissue samples were compensated with suitable post hoc tests.

## 5. Conclusions

Our findings confirm differences in matrix composition, remodeling, and a higher local inflammatory activity in the DDwoR diagnosis. Although no significant differences were identified between the DDwoR-subgroups, our findings indicate the importance of differentiating the subdiagnoses of DDwoR through further validation in larger cohorts. This study also highlights the need for further characterization of the synovial tissues to systemically describe the diagnoses from different aspects, including the basic histology and inflammatory profile.

## Figures and Tables

**Table 1:** Summary of the patient demographic data in relation to the diagnostic subgroups.

Patient characteristics	Number (%) of patients or mean when specified			
	DDwoR-SO (*n* = 10)	DDwoR-DO (*n* = 10)	DDwR (*n* = 9)	Total (*n* = 29)
Gender				
Female	10 (100)	9 (90)	6 (66)	25 (86)
Male	0 (0)	1 (10)	3 (33)	4 (14)
Age (years)				
Mean ± SD	46.5 ± 11.7	41.4 ± 20.3	43.1 ± 10.4	43.7 ± 14.6
(Range)	(32–63)	(20–68)	(27–55)	(20–68)
Previous trauma				
Yes	5 (50)	0 (0)	1 (11)	6 (21)
No	4 (40)	10 (100)	8 (89)	22 (76)
N.s.	1 (10)	0 (0)	0 (0)	1 (3)
Symptom duration (months)				
Mean ± SD	17.4 ± 10.6	11.9 ± 9.3	27.5 ± 28.7	18.4 ± 18.1
Type of operative intervention				
Arthroscopy	10 (100)	9 (90)	0 (0)	19 (66)
Discectomy	0 (0)	1 (10)	9 (100)	10 (34)
Operative intervention outcome				
Successful	4 (40)	6 (60)	5 (55)	15 (52)
Good	5 (50)	3 (30)	2 (22)	10 (34)
Intermediate	1 (10)	1 (10)	2 (22)	4 (14)
Deteriorated	0 (0)	0 (0)	0 (0)	0 (0)

Abbreviations: DO, delayed onset; DDwoR, discdisplacement without reduction; DDwR, disc displacement with reduction; N.s., not specified; SD, standard deviation; SO, sudden onset.

## Data Availability

The data used to support the findings of this study are available from the corresponding author upon request.

## Supporting Information

Supporting Information S1 Data 1: shows the quantification of histological and immunohistochemical staining performed using generalized linear models with a gamma distribution and presented as fold changes in mean pixel area ratio.

Supporting Information S2 Data 2: includes the negative controls for immunohistochemical staining.
