# Supplementary material for: Synovial Matrix Remodeling and Inflammatory Profile in Disc Displacement of the Temporomandibular Joint: An Observational Case-Control Study
Source: Int J Dent. 2024 Sep 18;2024:2450066. doi: 10.1155/2024/2450066 (PMC11424871; doi:10.1155/2024/2450066)
Supplement: Supporting Information S1 — Data 1: shows the quantification of histological and immunohistochemical staining performed using generalized linear models with a gamma distribution and presented as fold changes in mean pixel area ratio. [file 2450066.f1.pdf]

Synovial Matrix Remodelling and Inflammatory Profile in Disc Displacement of the Temporomandibular Joint: An Observational Case-Control Study

Pallavi Khattar, Mattias Ulmner, Henrike Häbel, Bodil Lund, Rachael Sugars

Supplementary Material (S1)

Quantification of fibrosis, and immunostaining of extracellular components and immune cell markers. Quantification of histological and immunohistochemical staining was performed using generalized linear models with a gamma distribution and presented as fold-changes in mean pixel area ratio. Tables show the pixel-area fold-changes with 95% confidence intervals with respect to the disc displacement diagnosis (disc displacement with reduction (DDwR), disc displacement without reduction (DDwoR) of delayed (DO) and sudden onset (SO). The outcomes were compared against the reference patient material, DDwR, which was set at 1. Pairwise comparisons between DDwoR-SO and DDwoR-DO, followed by Bonferroni correction, are also presented.

Statistical significance was taken with a P values **** = ≤ 0.001, *** = ≤ 0.005, ** = ≤ 0.01 and * = ≤ 0.05.

Van Gieson

Number of observations = 29

DDwR	Fold-change	Std. Err.	z	P>|z|	[95% Conf.	Interval]
DDwoR-DO	1.000	0.060	-0.00	1.000	0.888	1.126
DDwoR-SO	1.016	0.061	0.27	0.790	0.903	1.144
_cons	0.639	0.028	-10.19	0.000	0.587	0.697

	Delta-method	Bonferroni	
Pairwise comparisons	Str. Err.	z	P>|z|
DDwoR-DO vs DDwR	0.039	-0.00	1.000
DDwoR-SO vs DDwR	0.039	0.27	1.000
DDwoR-SO vs DDwoR-DO	0.038	0.27	1.000

Supplementary Material, S1

Collagen type I

Number of observations = 29

DDwR	Fold-change	Std. Err.	z	P>|z|	[95% Conf.	Interval]
DDwoR-DO	2.338	1.085	1.83	0.067	0.942	5.806
DDwoR-SO	0.942	0.437	-0.13	0.897	0.349	2.339
_cons	0.068	0.023	-8.00	0.000	0.035	0.131

	Delta-method	Bonferroni	
Pairwise comparisons	Str. Err.	z	P>|z|
DDwoR-DO vs DDwR	0.055	1.63	0.307
DDwoR-SO vs DDwR	0.031	-0.13	1.000
DDwoR-SO vs DDwoR-DO	0.054	-0.73	0.249

Collagen type III

Number of observations = 29

DDwR	Fold-change	Std. Err.	z	P>|z|	[95% Conf.	Interval]	
DDwoR-DO	6.162	2.700	4.15	0.000	2.610	14.546	****
DDwoR-SO	1.411	0.618	0.79	0.432	0.598	3.330	
_cons	0.004	0.001	-17.52	0.000	0.002	0.007	

	Delta-method	Bonferroni		
Pairwise comparisons	Str. Err.	z	P>|z|	
DDwoR-DO vs DDwR	0.007	2.74	0.019	*
DDwoR-SO vs DDwR	0.002	0.77	1.000	
DDwoR-SO vs DDwoR-DO	0.007	-2.49	0.038	*

Lumican

Number of observations = 29

DDwR	Fold-change	Std. Err.	z	P>|z|	[95% Conf.	Interval]	
DDwoR-DO	1.508	0.307	2.02	0.044	1.012	2.247	*
DDwoR-SO	1.015	0.207	0.07	0.942	0.681	1.513	
_cons	0.174	0.026	-11.84	0.000	0.130	0.232	

	Delta-method	Bonferroni	
Pairwise comparisons	Str. Err.	z	P>|z|
DDwoR-DO vs DDwR	0.045	1.97	0.147
DDwoR-SO vs DDwR	0.036	0.07	1.000
DDwoR-SO vs DDwoR-DO	0.044	-1.93	0.159

Supplementary Material, S1

Matrix metalloproteinase 2 (MMP-2)

Number of observations = 29

DDwR	Fold-change	Std. Err.	z	P>|z|	[95% Conf. Interval]		
DDwoR-DO	0.689	0.126	-2.04	0.042	0.481	0.986	*
DDwoR-SO	0.745	0.136	-1.61	0.107	0.520	1.066	
_cons	0.212	0.028	-11.66	0.000	0.164	0.275	

	Delta-method	Bonferroni	
Pairwise comparisons	Str. Err.	z	P>|z|
DDwoR-DO vs DDwR	0.034	-1.96	0.150
DDwoR-SO vs DDwR	0.035	-1.57	0.349
DDwoR-SO vs DDwoR-DO	0.027	0.44	1.000

Tissue inhibitor of metalloproteinases 2 (TIMP-2)

Number of observations = 29

DDwR	Fold-change	Std. Err.	z	P>|z|	[95% Conf. Interval]	
DDwoR-DO	0.486	0.212	-1.66	0.097	0.207	1.141
DDwoR-SO	0.481	0.209	-1.68	0.092	0.205	1.128
_cons	0.069	0.022	-8.45	0.000	0.037	0.129

	Delta-method	Bonferroni	
Pairwise comparisons	Str. Err.	z	P>|z|
DDwoR-DO vs DDwR	0.024	-1.48	0.418
DDwoR-SO vs DDwR	0.024	-1.50	0.404
DDwoR-SO vs DDwoR-DO	0.014	-0.03	1.000

Transforming growth factor beta-1 (TGF-β1)

Number of observations = 29

DDwR	Fold-change	Std. Err.	z	P>|z|	[95% Conf. Interval]	
DDwoR-DO	0.923	0.605	-0.12	0.903	0.255	3.338
DDwoR-SO	2.547	1.671	1.43	0.154	0.704	9.212
_cons	0.003	0.002	-11.94	0.000	0.001	0.009

	Delta-method	Bonferroni	
Pairwise comparisons	Str. Err.	z	P>|z|
DDwoR-DO vs DDwR	0.002	-0.12	1.000
DDwoR-SO vs DDwR	0.004	1.24	0.641
DDwoR-SO vs DDwoR-DO	0.004	1.33	0.552

Supplementary Material, S1

Transforming growth factor beta-3 (TGF- β3)

Number of observations = 29

DDwR	Fold-change	Std. Err.	z	P>|z|	[95% Conf.	Interval]
DDwoR-DO	1.758	0.995	1.01	0.312	0.587	5.327
DDwoR-SO	0.713	0.402	-0.60	0.548	0.237	2.150
_cons	0.002	0.001	-15.52	0.000	0.001	0.004

	Delta-method	Bonferroni	
Pairwise comparisons	Str. Err.	z	P>|z|
DDwoR-DO vs DDwR	0.00.	0.96	1.000
DDwoR-SO vs DDwR	0.001	-0.58	1.000
DDwoR-SO vs DDwoR-DO	0.001	-1.43	0.460

CD4

Number of observations = 29

DDwR	Fold-change	Std. Err.	z	P>|z|	[95% Conf.	Interval]	
DDwoR-DO	2.816	1.086	2.68	0.007	1.322	5.997	**
DDwoR-SO	5.227	2.016	4.29	0.000	2.454	11.133	****
_cons	0.004	0.001	-19.54	0.000	0.002	0.007	

	Delta-method	Bonferroni		
Pairwise comparisons	Str. Err.	z	P>|z|	
DDwoR-DO vs DDwR	0.003	2.27	0.069	
DDwoR-SO vs DDwR	0.006	2.99	0.008	**
DDwoR-SO vs DDwoR-DO	0.007	1.53	0.378	

CD68

Number of observations = 28

DDwR	Fold-change	Std. Err.	z	P>|z|	[95% Conf.	Interval]	
DDwoR-DO	4.585	1.823	3.83	0.000	2.103	9.994	****
DDwoR-SO	7.180	2.782	5.09	0.000	3.359	15.344	****
_cons	0.002	0.001	-21.40	0.000	0.001	0.004	

	Delta-method	Bonferroni		
Pairwise comparisons	Str. Err.	z	P>|z|	
DDwoR-DO vs DDwR	0.003	2.72	0.020	*
DDwoR-SO vs DDwR	0.005	3.19	0.004	***
DDwoR-SO vs DDwoR-DO	0.006	1.12	0.783	
