# Supplementary material for: Synovial Matrix Remodeling and Inflammatory Profile in Disc Displacement of the Temporomandibular Joint: An Observational Case-Control Study
Source: Int J Dent. 2024 Sep 18;2024:2450066. doi: 10.1155/2024/2450066 (PMC11424871; doi:10.1155/2024/2450066)

Synovial Matrix Remodelling and Inflammatory Profile in Disc Displacement of the Temporomandibular Joint: An Observational Case-Control Study

Pallavi Khattar, Mattias Ulmner, Henrike Häbel, Bodil Lund, Rachael Sugars

Supplementary Material (S2)

Negative controls for immunohistochemistry staining. Representative images of synovial tissue sections stained without primary antibody for immunohistochemistry (scale bar 20 µm).

Primary antibody omission control

Stained synovial tissue sections

Goat anti-mouse-IgG (TIMP 2, Coll III, CD68)

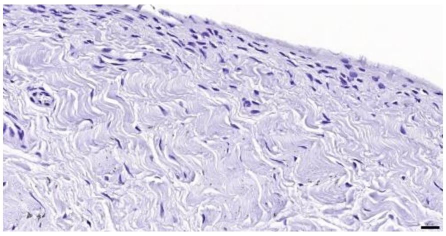

Goat anti-rabbit-IgG (CD4, Coll I, TGF-β1 and β3)

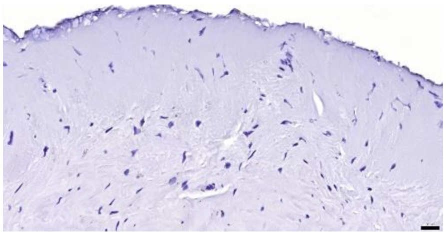

Rabbit anti-goat-IgG (Lumican, MMP-2)

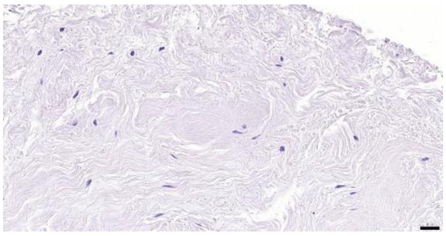

Supplement: Supporting Information S2 — Data 2: includes the negative controls for immunohistochemical staining. [file 2450066.f2.pdf]
